# Brand-specific estimates of influenza vaccine effectiveness for the 2021–2022 season in Europe: results from the DRIVE multi-stakeholder study platform

**DOI:** 10.3389/fpubh.2023.1195409

**Published:** 2023-07-20

**Authors:** Anke L. Stuurman, Antonio Carmona, Jorne Biccler, Alexandre Descamps, Miriam Levi, Ulrike Baum, Ainara Mira-Iglesias, Stefania Bellino, Uy Hoang, Simon de Lusignan, Roberto Bonaiuti, Bruno Lina, Caterina Rizzo, Hanna Nohynek, Javier Díez-Domingo, DRIVE Study Contributors, Anca Cristina Drăgănescu

**Affiliations:** ^1^P95 Epidemiology and Pharmacovigilance, Leuven, Belgium; ^2^Fundación para el Fomento de la Investigación Sanitaria y Biomédica de la Comunidad Valenciana (Fisabio), Valencia, Spain; ^3^Biomedical Research Consortium in Epidemiology and Public Health (CIBER-ESP), Instituto de Salud Carlos III, Madrid, Spain; ^4^Inserm CIC 1417, Assistance Publique Hopitaux de Paris (APHP), CIC Cochin-Pasteur, Paris, France; ^5^UFC Epidemiologia, Dipartimento di Prevenzione, Azienda USL Toscana Centro, Firenze, Italy; ^6^Department of Health Security, Finnish Institute for Health and Welfare, Helsinki, Finland; ^7^Department of Infectious Diseases, Istituto Superiore Di Sanità (ISS), Rome, Italy; ^8^Oxford-Royal College of General Practitioners Research and Surveillance Centre, Nuffield Department of Primary Care Health Sciences, University of Oxford, Oxford, United Kingdom; ^9^Department of Neurosciences, Psychology, Drug Research and Child Health, University of Florence, Firenze, Italy; ^10^VirPath Research Laboratory, International Center for Infectiology Research, University Claude Bernard Lyon, Lyon, France; ^11^Department of Translational Research and New Technologies in Medicine and Surgery, University of Pisa, Pisa, Italy

**Keywords:** vaccine effectiveness, influenza, influenza vaccines, test-negative design, post authorization, real-world evidence, Europe

## Abstract

**Introduction:**

Development of Robust and Innovative Vaccine Effectiveness (DRIVE) was a European public–private partnership (PPP) that aimed to provide annual, brand-specific estimates of influenza vaccine effectiveness (IVE) for regulatory and public health purposes. DRIVE was launched in 2017 under the umbrella of the Innovative Medicines Initiative (IMI) and conducted IVE studies from its pilot season in 2017–2018 to its final season in 2021–2022.

**Methods:**

In 2021–2022, DRIVE conducted four primary care-based test-negative design (TND) studies (Austria, Italy, Iceland, and England; involving >1,000 general practitioners), nine hospital-based TND studies (France, Iceland, Italy, Romania, and Spain, for a total of 21 hospitals), and one population-based cohort study in Finland. In the TND studies, patients with influenza-like illness (primary care) or severe acute respiratory infection (hospital) were enrolled, and laboratory tested for influenza using RT-PCR. Study contributor-specific IVE was calculated using logistic regression, adjusting for age, sex, and calendar time, and pooled by meta-analysis.

**Results:**

In 2021–2022, pooled confounder-adjusted influenza vaccine effectiveness (IVE) estimates against laboratory-confirmed influenza (LCI) overall and per type and subtype/lineage was produced, albeit with wide confidence intervals (CI). The limited circulation of influenza in Europe did not allow the network to reach the optimal sample size to produce precise IVE estimates for all the brands included. The most significant IVE estimates were 76% (95% CI 23%−93%) for any vaccine and 81% (22%−95%) for Vaxigrip Tetra in adults ≥65 years old and 64% (25%−83%) for Fluenz Tetra in children (TND primary care setting), 85% (12%−97%) for any vaccine in adults 18–64 years (TND hospital setting), and 38% (1%−62%) in children 6 months−6 years (population-based cohort, mixed setting).

**Discussion:**

Over five seasons, DRIVE collected data on >35,000 patients, more than 60 variables, and 13 influenza vaccines. DRIVE demonstrated that estimating brand-specific IVE across Europe is possible, but achieving sufficient sample size to obtain precise estimates for all relevant stratifications remains a challenge. Finally, DRIVE's network of study contributors and lessons learned have greatly contributed to the development of the COVID-19 vaccine effectiveness platform COVIDRIVE.

## Introduction

According to the European Center for Disease Prevention and Control (ECDC), seasonal influenza affects 4–50 million European citizens each year and is associated with 15,000–70,000 deaths annually, exerting a significant health and economic burden (1). Along with good hygiene, vaccination is considered the best action to protect against influenza; however, vaccine performance varies between influenza seasons. This performance is affected by multiple factors, such as the vaccine technology platform used, the match between the circulating and vaccine virus strains, and/or an individual's immune response. The above findings highlight the need for vaccine type, influenza strain, age-stratified analyses, and season-specific estimates of influenza vaccine effectiveness (IVE).

The Development of Robust and Innovative Vaccine Effectiveness (DRIVE) project is a public–private partnership that was conceived in 2017 as a proof of concept for the annual estimation of brand-specific IVE in Europe (2). DRIVE was a 5-year Innovative Medicines Initiative (IMI) project, equally funded by the European Commission and the European Federation of Pharmaceutical Industries and Associations (EFPIA). It was initiated in response to changes in the European Medicines Agency's (EMA) post-licensing requirements for influenza vaccines in Europe, which now stipulate the need for manufacturers to provide post-licensing brand-approval IVE estimates for their products.

**Figure 1:**
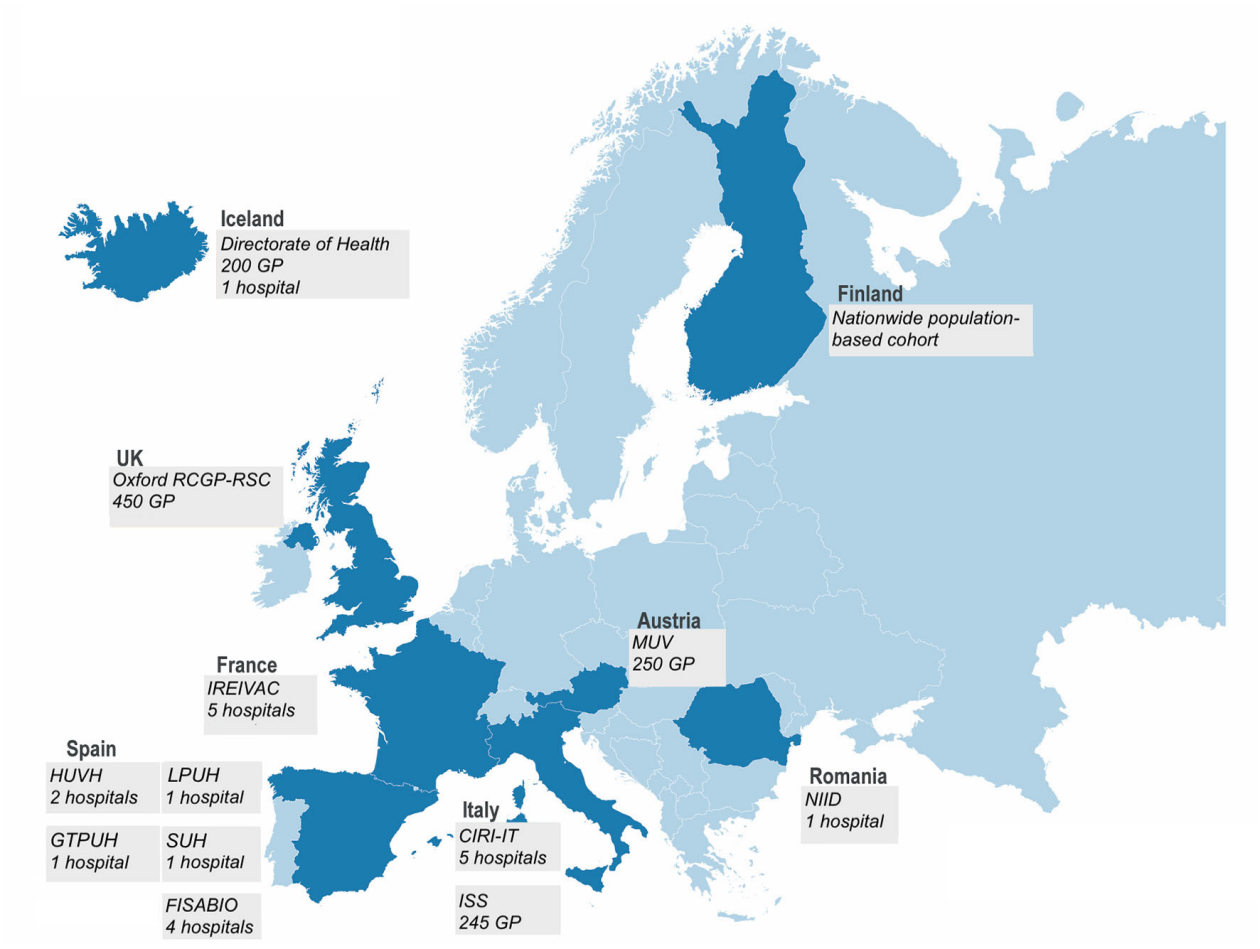
In the 2021–2022 season, the DRIVE network consisted of 12 independent study contributors in seven European countries conducting test-negative design (TND) studies and a population-based cohort study in Finland.

For the dual goal of addressing regulatory requirements and generating brand-specific IVE data for public health purposes, a multi-stakeholder public–private partnership of 16 partners from public health institutes, academia, small and medium-sized enterprises, and vaccine companies was established (2). The consortium managed to create a unique, large, and efficient study platform (Figure 1), as a large and geographically diverse network is required to obtain a sufficient sample size and to cover all vaccine brands in Europe.

In DRIVE, independently operating study contributors collected data and followed DRIVE core protocols for TND or population-based cohort studies (generic protocols are available on the DRIVE website at the URL: https://www.drive-eu.org/index.php/results/deliverables/), and a statistical analysis plan was defined upfront. Data from multiple study contributors enabled to increase in the sample size, geographic coverage, and the number of influenza vaccine brands included. Study contributors uploaded data and site-specific VE estimates were centrally calculated and subsequently pooled by meta-analysis. VE estimates were confounder-adjusted and stratified by age, setting, and influenza type/strain. In order to mitigate the risks of potential conflicts of interest by vaccine companies, study conduct, IVE analysis, and interpretation were conducted by public partners in the consortium and independent scientific oversight was ensured by the Independent Scientific Committee.

DRIVE has conducted IVE studies for five consecutive years: from its pilot season in 2017–2018 to the final season in 2021–2022. This manuscript presents the IVE results of the DRIVE study for the 2021–2022 season and an overview of the evolution of the DRIVE studies during its existence.

## Materials and methods

### Study design

#### TND studies

In the 2021–2022 season, TND studies were conducted in primary care (four networks, covering over 1,000 GPs) and hospital settings (five individual hospitals and four hospital networks, with a total of 21 hospitals) from seven European countries (Table 1, Figure 1). Swabs were collected from subjects presenting with influenza-like illness [ILI, European Center for Disease Prevention and Control (ECDC) case definition (3)] in the primary care setting and with a severe acute respiratory infection [SARI, I-MOVE+ 2017–2018 case definition (4)] in the hospital setting (except for the FISABIO hospital network and Iceland's hospital and GP network, where an alternative case definition was used—see Table 1). The swabs were tested for influenza using RT-PCR; influenza subtyping was performed for positive influenza samples. The samples were also routinely tested for SARS-CoV-2 using RT-PCR.

**Table 1:** Study contributors participating in the TND studies, 2021–2022 season.

**Country**	**Study contributor name**	Number of primary care physicians or hospitals where subjects with ILI/SARI^*^ were identified	**Population covered**
**Primary care**			
Austria	Medical University Vienna (MUV)	250	6 months−17 years; 18–64 years; ≥65 years
Iceland	Directorate of Health (EL GP)	Ca. 200^**^	6 months−17 years; 18–64 years; ≥65 years
Italy	Istituto Superiore di Sanità (ISS)	Ca. 245	6 months−17 years; 18–64 years; ≥65 years
England^***^	Royal College of General Practitioners (RCGP) Research and Surveillance Center (RSC) & University of Oxford	450	6 months−17 years; 18–64 years; ≥65 years
**Hospital**			
France	Innovative Clinical Research Network in Vaccinology (I-REIVAC)	5	18–64 years; ≥65 years
Iceland	Directorate of Health (EL-HOSP). Landspitali University Hospital	1^**^	6 months−17 years; 18–64 years; ≥65 years
Italy	Italian Hospital Network coordinated by Centro Interuniversitario di Ricerca sull'Influenza e le altre Infezioni Trasmissibili Italy (CIRI-IT BIVE)	5	18–64 years; ≥65 years
Romania	National Institute for Infectious Disease “Prof. Dr. Matei Balş” (NIID), Bucharest	1	18–64 years; ≥65 years
Spain	Fundación para el Fomento de la Investigación Sanitaria y Biomédica de la Comunitat Valenciana (FISABIO)	4	6 months−17 years; 18–64 years; ≥65 years
Spain	Hospital Universitario Germans Trias i Pujol (GTPUH), Badalona	1	18–64 years; ≥65 years
Spain	Hospital Universitario La Paz (LPUH), Madrid	1	18–64 years; ≥65 years
Spain	Hospital Universitario de Salamanca (SUH), Salamanca	1	18–64 years; ≥65 years
Spain	Hospital Universitari Vall d'Hebron (HUVH), Barcelona and Hospital Universitari de Girona Doctor Josep Trueta (HUJT), Girona	2	18–64 years; ≥65 years

^*^Alternative case definitions were used for Spain FISABIO and Iceland EL GP/EL HOSP. FISABIO: Case definition children <5 years: hospitalization for any acute respiratory reason with symptom onset (of any symptom possibly related to influenza: acute upper and lower respiratory disease; dyspnea, breath anomalies, shortness of breath, tachypnea; asthma; pneumonia and influenza; fever or fever of unknown or non-specified origin; cough; apnea; COVID-19 or suspected COVID-19) in the 7 days prior to admission. Case definition ≥5 years: modified SARI case definition where only symptoms before admission are considered, “deterioration of general condition” is not considered, and symptoms could not have started more than 7 days before the date of admission instead of the date of sampling. Iceland: presenting with respiratory tract infection (not further defined).

^**^All samples sent to the national reference laboratory.

^***^RCGP-RSC data were not included in the analysis as no influenza cases were confirmed for the 2021–2022 season.

ILI, influenza-like illness; SARI, severe acute respiratory infection.

#### Population-based cohort study

One population-based cohort study was conducted by the Finnish Institute for Health and Welfare (THL) in Finland by linking data from five national registers through personal identifiers.

### Study period

For TND studies, the start of the study period for IVE analyses for the DRIVE studies was defined as the first week of two consecutive weeks when influenza viruses were detected at the study contributor level (based on the data provided to DRIVE), and the end as the week prior to the first of two consecutive weeks when no influenza viruses were detected at the study contributor level (based on the data provided to DRIVE) or 30 April 2022, whichever occurred first. In the population-based cohort study, the study period was defined *a priori* from 4 October 2021 to 30 April 2022.

In 2021–2022, a bimodal influenza season was observed, with different influenza peaks: a smaller one in December 2021–January 2022 and a larger one in March–April 2022. As a consequence, three scenarios with respect to the study period for analysis were observed: (i) the first peak did not lead to enough cases to trigger the study start definition but only the second peak did (e.g., Italy CIRI-IT BIVE); (ii) the first peak triggered the study start definition and the occasional influenza cases observed between the first and the second peaks ensured that the study end definition was not triggered before the second peak (e.g., FISABIO); and (iii) there was a very minor first peak that triggered the study start definition with no cases until the second wave started; in this case, only the second wave was considered (e.g., NIID).

### Study population

For the TND studies, the study population consisted of non-institutionalized subjects ≥6 months of age with no contraindication for influenza vaccination, no prior positive influenza test in the same season, and a swab taken <8 days after ILI/SARI onset. In hospital settings, subjects hospitalized <48 h prior to symptom onset or with symptom onset ≥48 h after hospital admission were excluded. For each subject, data on covariates (at least age, sex, and date of symptom onset), vaccination status, and vaccine brands for vaccinated subjects were collected. Cases and controls were considered fully vaccinated if they had received seasonal influenza vaccination >14 days before the start of the ILI/SARI episode.

For the population-based cohort, the study population consisted of all registered Finnish residents aged 6 months−6 years and 65–100 years. The case definition was laboratory-confirmed influenza, as registered in the National Infectious Diseases Register. Using the Care Register for Health Care, it was possible to identify laboratory-confirmed influenza cases who were inpatients for any reason on the day of laboratory confirmation.

### Statistical methods

Study contributor-specific IVE and 95% confidence intervals (CIs) for TND studies were calculated using logistic regression as IVE = (1 – OR) × 100%, where OR is the odds ratio comparing the odds of influenza among vaccinated and unvaccinated subjects. As supported by an exploratory analysis of the 2018–2019 and 2019–2020 DRIVE datasets, a parsimonious confounder adjustment was performed, which included sex, a smooth function of age, and a smooth function of the calendar week or date of symptom onset (5).

Study contributor-specific IVE estimates from the TND studies were pooled through a random-effects meta-analysis, which assumes that the observed effect estimates can vary across study sites because of differences in the treatment effect in each study site (e.g., due to differences in population, healthcare utilization, circulating influenza strains) in addition to sampling variability (6, 7). Study contributor-specific IVE estimates that were both outlying (studentized deleted residuals |*r*| >2.5) and influential (standardized |DFBETAs| >2/√*n*) were excluded from the pooled analysis; a sensitivity analysis including these estimates was conducted.

In the present study, we focused our attention on significant IVE estimates and those with a CI width <40% (if any), although for the sake of transparency, the rest of the IVE estimates can be consulted in the DRIVE 2021–2022 results report and the WebAnnex.

For the population-based cohort study, contributor-specific semi-crude estimates (adjusted only for calendar time) and confounder-adjusted estimates (adjusted as in the TND studies) were obtained. IVE and 95% CIs were estimated using Poisson regression. IVE was calculated as IVE = (1 – IRR) × 100%, where IRR is the incidence rate ratio comparing the incidence of influenza in vaccinated subjects to the incidence of influenza in unvaccinated subjects.

### Ethical considerations

Each local study was approved by national, regional, or institutional ethics committees, as appropriate. All the studies were approved without the need for protocol revision; therefore, ethics committee approval was given in due time for the start of the DRIVE study. Written informed consent was obtained in two-thirds of the participating study sites; in the remaining one-third of study sites (i.e., THL, MUV, GTPUH, EL, HVUH), informed consent was not needed, as the DRIVE study was nested within the national/regional influenza surveillance systems.

## Results

### Influenza vaccines in the 2021–2022 season

A total of 12 influenza vaccines were licensed and marketed in the EU/European Economic Area (EEA)/UK for the season 2021–2022, and eight of these vaccines were captured in the DRIVE dataset. Details on vaccine characteristics, the approved age indication, and, for each age group, the countries that reported the vaccine brand in the 2021–2022 studies are listed in Table 2.

**Table 2:** Influenza vaccine characteristics by vaccine brand in the 2021–2022 season—vaccines licensed and marketed in Europe.

**Vaccine brand**	**Manufacturer**	**Valency**	**Type**	**Adjuvant**	**Culture**	**HA antigen content**	**Approved age indication**	**Countries (regions) where the vaccine brand was observed in the DRIVE dataset, by age group**		
								**6 months**−**17 years**	**18–64 years**	≥**65 years**
Afluria Tetra^a^	Seqirus	4	I	Non-Adj	Egg	SD	≥18 years	–	–	–
Chiroflu	Seqirus	3	I	Non-Adj	Egg	SD	≥6 months	–	–	–
Efluelda^ab^	Sanofi	4	I	Non-Adj	Egg	HD	≥60 years	n/a	AU	IT, SP (CT), AU, FR
Fluad^b^	Seqirus	3	I	Adj	Egg	SD	≥65 years	n/a	n/a	SP (V, M, CL), IT
Fluad Tetra^ab^	Seqirus	4	I	Adj	Egg	SD	≥65 years	n/a	n/a	IT, SP (CT, CL), AU
Fluarix Tetra^b^	GSK	4	I	Non-Adj	Egg	SD	≥6 months	IT, AU	IT, AU	IT, AU
Flucelvax Tetra^b^	Seqirus	4	I	Non-Adj	Cell (M)	SD	≥2 years	–	IT, AU, SP (V)	IT, AU, SP (V)
Fluenz Tetra^b^	AstraZeneca	4	LA	Non-Adj	Egg	SD	2–17 years	IT, AU, FI	n/a	n/a
Influvac	Abbott	3	I	Non-Adj	Egg	SD	≥6 months	–	–	–
Influvac Tetra^b^	Abbott	4	I	Non-Adj	Egg	SD	≥6 months	AU, SP (V)	AU, SP (V, CT), RO, FR	AU, SP (V, CT), ICE, FR
Supemtek^a^	Sanofi	4	R	Non-Adj	Cell (In)	SD	≥18 years	–	–	–
Vaxigrip Tetra^b^	Sanofi	4	I	Non-Adj	Egg	SD	≥6 months	ICE, IT, AU, FI, RO	ICE, IT, AU, FR, RO, SP (CT, M, CL)	ICE, IT, AU, FR, SP (CT), FI

^a^Newly available in the EU/EEA/UK in the 2021–2022 season.

^b^Influenza vaccines reported in the DRIVE database for the 2021–2022 season.

Adj, adjuvanted; AU, Austria; FI, Finland; FR, France; HD, high dose; I, inactivated; ICE, Iceland; In, insect cells; IT, Italy; LA, live attenuated; M, mammalian cells; Non-Adj, non-adjuvanted; R, recombinant; RO, Romania; SD, standard dose; SP, Spain (CT, Catalonia; CL, Castilla y León; M, Madrid; V, Valencia).

The recommended composition of influenza vaccines in the Northern Hemisphere for the 2021–2022 season can be consulted at the World Health Organization website (8).

### Influenza epidemiology in the 2021–2022 season

The circulation of influenza was relatively low, and co-circulation of influenza and SARS-CoV-2 was observed. In most of the participating countries, the influenza season initially overlapped with the SARS-CoV-2 Omicron wave (from November 2021 to February 2022) and presented two peaks of higher activity: the first one in December 2021–January 2022 (peaking in week 52/2021) and a higher and unusually late peak in March–April 2022 (peaking between weeks 10 and 15 of 2022), which declined until May 2022 (9, 10).

Within the 2021–2022 DRIVE study, a total of 1,039 influenza cases were included in the analysis for the TND studies and 331 in the population-based cohort study. The number of influenza A cases exceeded the number of influenza B cases at all sites. Among influenza A cases with a known subtype, the most frequently identified subtype was A(H3N2) at all the participating sites (ranging from 47 to 100% of the subtyped viruses), in line with the data reported by the ECDC (9). Influenza B was only detected in Austria (0.6% of their influenza cases) and Finland (7%). All B cases included were of B/Victoria lineage, and no cases of B/Yamagata lineage were identified (11). Supplementary Table 1 describes the distribution of influenza cases by type and subtype for each study contributor.

The predominant A(H3N2) subclade was 3C.2a1b.2a.2, with a haemagglutinin (HA), with several substitutions that made it antigenically different from the A(H3N2) Cambodia lineage (3C.2a1b.2a.1) included in the 2021–2022 influenza vaccine. Therefore, a mismatch between the circulating A(H3N2) influenza strain and the strain included in that season's vaccine composition was observed.

### Subject and exposure characteristics for the 2021–2022 season

#### TND studies

In total, 1,039 cases and 5,255 controls of all ages were included in the main analysis of the TND studies, with 411 cases and 2,805 controls retained for analysis in the primary care setting and 628 cases and 2,450 controls in the hospital setting (Table 3; see Supplementary Figure 1 for attrition diagrams). The highest proportion of vaccinated controls was observed in the age group ≥65 years (33% in the primary care setting and 56% in the hospital setting). Subject characteristics by age group, setting, participating study contributors, and influenza vaccine brand are available in WebAnnex. The attrition diagrams by setting can be found in Supplementary Figure 1.

**Table 3:** Number and characteristics of cases and controls, median age, sex, and presence of chronic conditions results retained for analysis by study setting and age category, TND studies, 2021–2022.

	**Primary care**			**Hospital**		
	**6 months**−**17 years**	**18–64 years**	≥**65 years**	**6 months**−**17 years**	**18–64 years**	≥**65 years**
Cases [PV (%)]	189 (11)	181 (18)	41 (41)	211 (4)	206 (14)	211 (65)
Controls [PV (%)]	1,106 (16)	1,448 (13)	251 (33)	589 (6)	677 (22)	1,184 (56)
Age [mean (SD)]	6.57 (5.22)	39.18 (12.81)	76.03 (8.32)	4.42 (4.65)	45.49 (13.94)	79.34 (8.51)
**Sex**						
Female [*n* (%)]	610 (47.1)	934 (57.3)	172 (58.9)	369 (46.1)	443 (50.2)	626 (44.9)
**Presence of chronic conditions**						
At least one [*n* (%)]	82 (6.3)	379 (23.3)	165 (56.5)	86 (10.8)	394 (44.6)	1,002 (71.8)
Not available [*n* (%)]	106 (8.2)	281 (17.2)	67 (22.9)	598 (74.8)	280 (31.7)	328 (23.5)

Hosp, hospital; n, number; PC, primary care; PV, proportion vaccinated.

**Figure 2:**
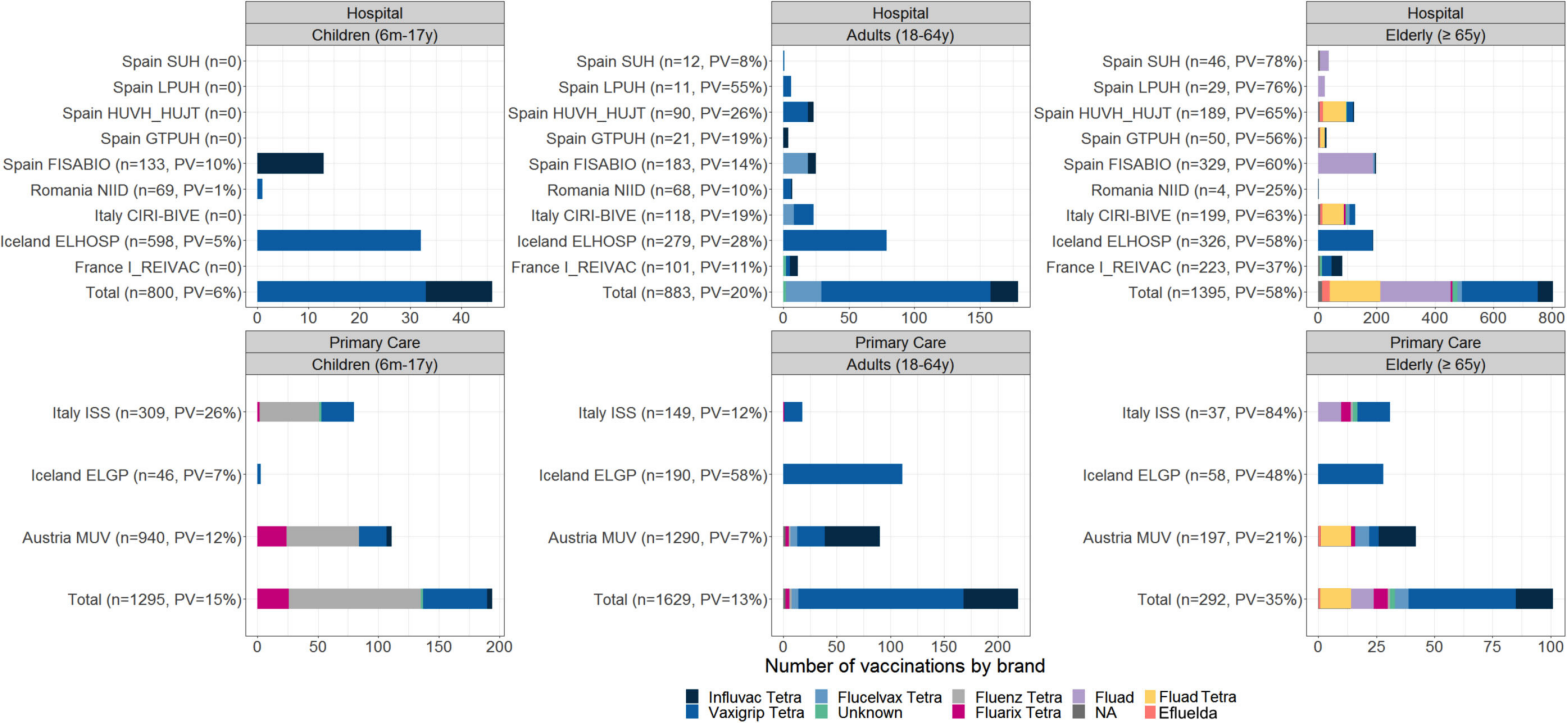
Number of vaccinated subjects among enrolled subjects and distribution of vaccine brands; TND studies, 2021–2022.

Age-specific brand distribution among vaccinated subjects by study contributor is shown in Figure 2. The most frequently reported vaccine brand across all age groups (6 months−17 years, 18–64 years, and ≥65 years) was Vaxigrip Tetra. In children from 6 months to 17 years, Vaxigrip Tetra was followed by Fluenz Tetra; in adult subjects 18–64 and ≥65 years, the second most reported vaccines were Influvac Tetra and Fluad, respectively. The majority of reported vaccine types among vaccinated subjects were as follows: inactivated quadrivalent egg-based and live-attenuated trivalent in children (54 and 46%, respectively); inactivated quadrivalent egg-based in adults 18–64 years (90%); adjuvanted inactivated trivalent or quadrivalent vaccines (51%); and quadrivalent egg-based vaccine (43% in adults ≥65 years; Figure 2). The brands that were not identified were Afluria Tetra (Seqirus), Chiroflu (Seqirus), Influvac (Abbott), and Supemtek (Sanofi).

The percentage of cases and controls with a positive SARS-CoV-2 test (among those tested for SARS-CoV-2) is shown in Supplementary Table 2. The proportion of subjects with a SARS-CoV-2 infection was highest among adults 18–64 years (39% in the primary care setting and 20% in the hospital setting) and those aged ≥65 years (46 and 19%, respectively). A total of 29 coinfections of influenza and SARS-CoV-2 were identified.

#### Population-based cohort study

In the population-based cohort, 169,823 person-years were available for analysis in the age group 6 months−6 years and 666,799 person-years in the age group ≥65 years. In Finland, during the 2021–2022 season, children were vaccinated with either Fluenz Tetra (recommended for children from 2 to 6 years) or Vaxigrip Tetra (recommended for children from 6 months to 6 years), while older adults were vaccinated with Vaxigrip Tetra (Table 4). The distribution of key covariates among exposed and unexposed individuals is shown in Supplementary Figure 2.

**Table 4:** Number of laboratory-confirmed influenza infections and person-years by vaccination status and vaccine for the population-based cohort study (Finland), 2021–2022.

**Analysis**	**Age group**	**Vaccinated**			**Unvaccinated**		
		**Number of influenza infections (mixed setting)**	**Number of hospitalized influenza infections** ^*^	**Person-years**	**Number of influenza infections**	**Number of hospitalized influenza infections** ^*^	**Person-years**
Any vaccine	6 months−6 years	29	8	37,508	93	17	132,315
	≥65 years	118	51	304,570	91	41	362,229
Vaxigrip tetra	6 months−6 years	6	1	8,747	93	17	132,315
	≥65 years	118	51	300,590	91	41	362,229
Fluenz Tetra	2–6 years	22	7	28,508	79	16	109,650

^*^Infections timely associated with hospitalization.

py, person-years; unvac, unvaccinated; vac, vaccinated.

### Overall and brand-specific IVE pooled confounder-adjusted estimates for the 2021–2022 season

#### TND studies

**Figure 3:**
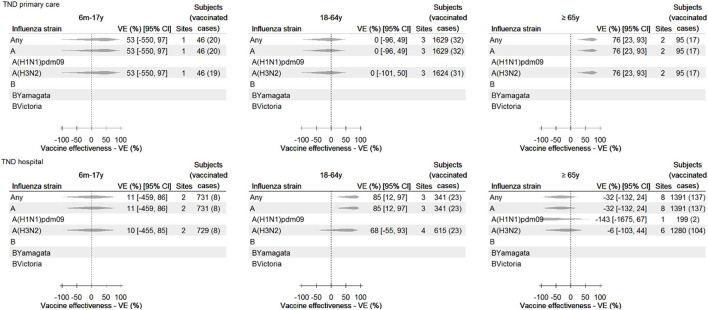
Any influenza vaccine: pooled confounder-adjusted (age, sex, and date of symptom onset) influenza vaccine effectiveness against laboratory-confirmed influenza, overall and by type and subtype/lineage, by setting and age group, 2021–2022—TND studies. For some study contributors, IVE could not be calculated (e.g., due to a value of 0 in the 2 × 2 table), and/or the estimate was both outlying and influential (and therefore excluded). Consequently, data from these sites are not included in the pooled estimate, and therefore, the number of subjects in this figure may be lower than described in Table 3.

The pooled confounder-adjusted IVE estimates **for each vaccine**, stratified by age group and healthcare setting, are provided in Figure 3. All estimates had wide CIs. Significant IVE estimates were obtained in the primary care setting for any influenza vaccine among those aged ≥65 years [VE 76% (95% CI 23–93) for those aged ≥65 years, against any influenza and type A and subtype A(H3N2)] and in the hospital setting for any influenza vaccine among adults aged 18–64 years [(VE 85% [95% CI 12–97]) against any influenza and influenza A type].

**Brand-specific** vaccine effectiveness estimates against any influenza are shown in Supplementary Table 3. Brand-specific estimates were available for at least one age/setting stratum for Efluelda, Fluad, Fluad Tetra, Fluarix Tetra, Flucelvax Tetra, Fluenz Tetra, Influvac Tetra, and Vaxigrip Tetra. However, due to the low sample size for most brands and strata, the majority of brand-specific estimates had very wide CIs. Significant brand-specific IVE estimates were obtained in older adults in the primary care setting, with VE for Vaxigrip Tetra against influenza of 81% (95% CI 22–95) and in children in the primary care setting, with VE for Fluenz Tetra against any influenza of 64% (95% CI 25–83). All pooled crude estimates, pooled confounder-adjusted IVE estimates by influenza vaccine brand (stratified by age group and setting), and adjusted study contributor-specific estimates can be consulted in the DRIVE 2021–2022 results report and the WebAnnex.

Several sensitivity analyses were performed and are available in the DRIVE 2021–2022 results report and the WebAnnex. For instance, excluding subjects with a time between ILI/SARI onset and swab ≥4 days led to decreases in the VE point estimates against any influenza among children (absolute Δ −5%) and adults (Δ −71%) but did not affect the estimates obtained among older adults. In the hospital setting, such a restriction led to increases among children (Δ +30%) and older adults (Δ +55%) and decreases among adults (Δ −6%). Additionally, including study contributor-specific estimates for any vaccine and any influenza that was both outlying and influential resulted in the pooling of two additional estimates for children in the primary care setting [the pooled IVE including these estimates was 48% (95% CI −142 to 89)], one additional estimate for older adults in the primary care setting [32% (95% CI −6,008 to 99)], and one additional estimate for adults in the hospital setting [65% (95% CI −129 to 95)]. Finally, due to the low number of SARS-CoV-2 and influenza coinfections identified (*n* = 29) across all age groups/settings, no sensitivity analysis stratified by SARS-CoV-2 status was conducted.

#### Population-based cohort study

Overall and brand-specific confounder-adjusted pooled IVE estimates against influenza are shown in Table 5 and Supplementary Table 4, respectively. A statistically significant overall IVE estimate was obtained in the mixed setting for children 6 months to 6 years against laboratory-confirmed influenza A [38% (95% CI 1–62)]. Furthermore, IVE for Vaxigrip Tetra against any laboratory-confirmed influenza in older adults was estimated at 15% (95% CI −12% to 36%) and was similar for influenza A and any influenza in the hospital setting.

**Table 5:** Confounder-adjusted influenza vaccine effectiveness of any vaccine and by vaccine brand against any influenza and influenza A for the Finnish population-based cohort, mixed setting, and hospital setting, 2021–2022.

	**Any influenza IVE % [95% CI]**	**Influenza A IVE % [95% CI]**
**Mixed setting**		
**6 months**−**6 years**		
Any vaccine	23 [−17, 50]	38 [1, 62]
≥**65 years**		
Any vaccine	16 [−11, 36]	14 [−14, 35]
**Hospital setting**		
**6 months**−**6 years**		
Any vaccine	−14 [−169, 52]	–
≥**65 years**		
Any vaccine	17 [−26, 45]	–

The IVE estimates for influenza B are not displayed as <10 influenza B cases were reported. However, all crude and confounder-adjusted IVE estimates by influenza vaccine brand, stratified by age group and setting, can be consulted in the DRIVE 2021–2022 results report and the WebAnnex.

## Discussion

### DRIVE 2021–2022 season IVE estimations

A total of 12 influenza vaccine brands were marketed in the EU/EEA/UK for the 2021–2022 season, eight of which were included in the DRIVE dataset, highlighting the ability of the DRIVE study network to cover a representative sample of the European influenza vaccine market. All except one of the vaccines observed were quadrivalent, reflecting the transition from trivalent to quadrivalent vaccines. The two most frequently observed vaccine brands per age group were the same as for the 2020–2021 season (12). The brands that were not identified were Afluria Tetra (Seqirus), Chiroflu (Seqirus), Influvac (Abbott), and Supemtek (Sanofi; Table 2). Afluria Tetra was only distributed in Germany, a country not included in DRIVE, and Supemtek was only available in the UK, where only a very limited number of subjects were enrolled. Chiroflu and Influvac are both trivalent vaccines that are being phased out. The inclusion of study contributors that together capture a sufficiently diverse range of vaccine availability is a particular challenge for a network aiming to study brand-specific IVE.

DRIVE conducted TND studies to produce pooled, confounder-adjusted influenza vaccine effectiveness estimates against LCI overall and per type and subtype/lineage, by setting and age group. The most significant overall and brand-specific IVE estimates showed a protective effect and were obtained among older adults in the primary care setting and adults in the hospital setting for any influenza and influenza A types. Despite CDC's recommendation to not report VE estimates if the CI exceeds 50% in order to avoid reporting uninterpretable estimates (13), DRIVE partners decided to report the most significant overall and brand-specific VE estimates even though their CI exceeded 50%. The less significant VE estimates are also available in the DRIVE 2021–2022 results report and the WebAnnex for full transparency of the analysis performed.

The population-based cohort study in Finland generated IVE estimates for the two influenza vaccine brands used in Finland and overall IVE. Due to the small number of influenza cases in Finland, all IVE estimates presented a wide CI and, therefore, were not informative. Interestingly, the two different settings, the hospital setting and the mixed primary care and hospital setting yielded similar results, which is in line with a review by Feng et al. (14).

### Findings from other European IVE studies in 2021–2022

To the best of our knowledge, DRIVE is the only multi-country IVE study in Europe that generates brand-specific IVE. Nevertheless, several studies focusing on overall IVE estimates for the 2021–2022 season were identified.

From 2007–2008 until 2021–2022, the I-MOVE (Influenza Monitoring Vaccine Effectiveness in Europe) consortium (15, 16) has conducted, in collaboration with the ECDC, annual studies to evaluate the effectiveness and impact of the influenza vaccines in Europe. The primary care-based I-MOVE study produced overall IVE estimates from 10 primary care study sites between October 2021 and mid-May 2022 (17). I-MOVE estimates among adults ≥65 years were 26% (95% CI −22 to 55) against influenza A(H3N2) and 23% (95% CI −21 to 51) against any influenza A, whereas, for DRIVE, the IVE estimate at the primary care setting for ≥65 years was 76% (95% CI 23–93) against any influenza A and A(H3N2). While the point estimates obtained from DRIVE and I-MOVE were different, the 95% of CIs overlapped, preventing meaningful comparisons.

Moreover, in children aged 2–6 years in Denmark, VE was estimated at 64.2% (95% CI 50.5–74.1) against non-hospitalized influenza A and 63% (95% CI 10.9–84.4) against hospitalized influenza A; 92% of vaccinated children had received the live-attenuated influenza vaccine (LAIV) (18). In the UK, the VE of LAIV against influenza requiring an emergency department visit was 72% (95% CI 50–85) (19). In DRIVE, the point estimates for LAIV were 63% (95% CI 23–82) against influenza A in the TND primary care studies and 40% (95% CI −2.9 to 64.5) against any influenza in any setting among children 2–6 years in the Finnish population-based cohort study. Significant comparisons across these studies are hindered due to variations in study design, the outcomes examined, and the overlap in the 95% CIs of the point estimates.

Finally, VE against all-age outpatient influenza was estimated at 50% (95% CI 14–71) for any influenza and 31% (95% CI −29 to 64) for A(H3N2) in France (20); and VE against laboratory-confirmed influenza was estimated at 47% (CI not reported) for adults ≥65 years in Sweden (mixed inpatient and outpatient setting) (21), which is higher than the point estimates for this age group in DRIVE.

The discrepancies between the DRIVE estimates and those obtained from different studies may be partially explained by the differences among the participating countries in each study (different vaccine recommendations and virus circulation per country) and study settings. In addition, most of the abovementioned VE estimates had large CIs, so comparison across studies is complex. These caveats underline the necessity of collaboration between VE platforms and the harmonization of the methods used for larger and more representative European studies.

### Limitations

The sample size required for brand-specific IVE studies is large, due to the numerous vaccine brands and the multiple stratifications required. Given the limitations in sample size, overall and brand-specific estimates produced by DRIVE had wide confidence intervals, limiting their potential to be informative. In addition, the COVID-19 pandemic led to reduced influenza virus circulation, a shift in interest, and an overload of staff dedicated to the studies. Furthermore, the inability of potential collaborators to collect information on influenza vaccine brands, the presence of other overlapping VE networks, and the hesitancy toward the public–private governance model perceived in certain sectors of public health institutions were also limiting factors.

Other than the sample size, multiple factors affect the precision of the estimates, such as vaccination coverage and influenza attack rate, but also the true IVE, test sensitivity and specificity, statistical methods, and the heterogeneity of study contributor-specific IVE estimates. Given the width of the CIs of the majority of the IVE estimates generated in the 2021–2022 season, these should be interpreted with caution.

The availability of information on the presence of chronic conditions was limited for most of the (vaccinated) subjects and did not allow a solid assessment of the impact of additional adjustment for this variable. However, in an exploratory analysis of previous DRIVE data, additionally adjusting for the presence of at least one chronic condition did not have an important impact on VE estimates (5). Furthermore, given the low number of coinfections with SARS-CoV-2 (*n* = 29), no sensitivity analysis stratified by SARS-CoV-2 status was conducted.

In addition, VE may change during the influenza season due to waning immunity, but we could not study this in DRIVE due to the limited sample size.

Due to the observational nature of the studies, selection bias cannot be ruled out. In TND studies, the relationship between influenza vaccination and testing positive for influenza among those who are tested is studied. The use of a TND, therefore, reduces confounding due to healthcare-seeking behavior but introduces selection bias and may reduce the generalizability to the general population (22).

Healthcare-seeking behavior has changed during the COVID-19 pandemic, through different periods of the pandemic, and the changes are probably not uniform across countries. Therefore, it is not clear how this affects the TND and the subsequent IVE estimates. Finally, residual confounding may be present due to measurement errors in the confounders and other unmeasured confounders.

### Evolution of DRIVE studies through a multi-stakeholder platform (2017–2022)

DRIVE started with a pilot season (2017–2018) to establish the multi-country platform for TND and cohort studies using a limited number of sites (Table 6). The initial study contributors participated with data collected using their own protocols (23). As of 2018–2019, generic protocols were implemented by all sites and, along with the statistical analysis plan, were iteratively improved each season. An IT platform where study contributors could perform data quality checks and upload their anonymized dataset to a secure environment and where data were centrally analyzed was developed. The size of the study network increased from five sites in four countries in 2017/2018 to 13 sites in eight countries in 2021–2022. As of 2018–2019 (24), the network was sufficiently geographically diverse to capture the majority of influenza vaccine brands marketed in Europe (Table 6). From the 2018/2019 season onwards, DRIVE also supported the implementation and assessed the feasibility of using point-of-care PCR testing (POCT) to improve sampling numbers from primary care in England (25–27).

**Table 6:** Evolution of DRIVE studies from 2017–2018 to 2021–2022 influenza seasons.

**Influenza season**	**2017–2018**	**2018–2019**	**2019–2020**	**2020–2021**	**2021–2022**
Characteristics	High influenza circulation	Moderate influenza circulation	Moderate influenza circulation—study capped by COVID-19 emergence	No influenza circulation—COVID-19 pandemic	Very low influenza circulation—late influenza epidemic peak (March–April 2022) Omicron COVID-19 pandemic
Study network	5 study contributors 4 countries +950 GP 4 hospitals	10 study contributors 7 countries 377 GP 12 hospitals	14 study contributors 8 countries 388 GP 19 hospitals	14 study contributors 8 countries +500 GP 25 hospitals	13 study contributors 8 countries +1,000 GP 21 hospitals
Number of subjects	5,475 (TND) 288,655 py cohort Finland	9,351 (TND) 768,414 py cohort Finland	9,077 (TND) 511,854 py cohort Finland	7,025 (TND) 857,095 py cohort Finland	6,315 (TND) 836,622 py for cohort Finland
Number of LCI	2,844 (TND) 13,300 (cohort Finland)	3,339 (TND) 6,379 (cohort Finland)	3,500 (TND) >2,400 (cohort Finland)	4 (TND) 25 (cohort Finland)	1,039 (TND) 331 (cohort Finland)
Brand-specific IVE estimates (brands captured/brands marketed in EU/EEA/UK)	4/11	7/10	8/11 4 precise^*^ brand-specific IVE estimates	Did not reach threshold to trigger IVE estimation	8/12

GP, general practitioner; py, person-years; LCI, laboratory-confirmed influenza; py: person-years; TND, test-negative design.

^*^Precise: Brand-specific IVE estimates below the threshold of confidence interval width <40%, arbitrarily agreed upon by DRIVE researchers.

As a result of a *post hoc* analysis based on the 2018–2019 data (24), and as recommended by Lane et al. (28), a parsimonious set of confounders was defined to simplify the analysis. This approach is also supported by a recent exploratory analysis conducted with DRIVE data (5). The reduced set of confounders was used in the DRIVE studies from the 2019–2020 season and allowed the participation of study contributors with limited data on confounders, in addition to limiting the data discarded due to missing values and avoiding potential over-adjustment. Suggested confounding variables for the main analysis included age, sex, and calendar time, but sensitivity analyses including all potential confounders available (presence of chronic disease, number of hospitalizations and GP visits in the past 12 months, pregnancy) were also performed.

Throughout the existence of DRIVE, attaining a sufficient sample size to obtain meaningful estimates for brand-specific estimates, with their relevant strata, has been a challenge. DRIVE aimed to obtain precise estimates, defined as a CI width of <40%, although the level of precision required for decision-making by regulators and public health bodies remains unclear. Precise brand-specific estimates were obtained for Vaxigrip Tetra, Fluarix Tetra, and Fluad in the 2019/20 TND studies and for Fluenz Tetra and Vaxigrip Tetra in 2018–2019 and 2019–2020 in the population-based cohort (24, 29).

The relatively low influenza circulation, as a consequence of the non-pharmaceutical interventions implemented to fight the COVID-19 pandemic and the shift of attention and resources to COVID-19, largely impacted the 2020–2021 and 2021–2022 seasons, obstructing DRIVE from generating robust brand-specific IVE estimates in its last two seasons (30). The DRIVE study platform was adjusted to the COVID-19 pandemic context, and the core protocols and SAP were tailored with the support of the DRIVE partners, study contributors, and the Independent Scientific Committee. Furthermore, new variables related to SARS-CoV-2 testing, infection, treatment, and vaccination were collected.

Finally, the DRIVE infrastructure, study network, and governance model were leveraged to launch COVIDRIVE in 2021, a public–private partnership that currently brings together 12 partners and aims to conduct multi-country European studies to monitor brand-specific COVID-19 vaccine effectiveness (CVE) in real-world conditions (31). The COVIDRIVE partnership was set up in only 9 months thanks to the existence of the DRIVE study platform and partner collaborations. As of February 2023, the COVIDRIVE study is active in 15 hospitals (Belgium, Austria, Italy, and Spain), which have enrolled over 7,000 SARI patients and more than 3,000 COVID-19 cases.

## Conclusions

In a season marked by a relatively low influenza circulation in Europe, DRIVE was able to conduct its final study for brand-specific IVE evaluation in Europe. DRIVE obtained brand-specific IVE estimates for eight of the 12 influenza vaccine brands marketed in the EU/EEA/UK in the 2021–2022 season and generated brand-specific IVE estimations in a challenging environment. However, the majority of the IVE estimates had wide CIs and consequently must be interpreted with caution.

Through the five seasons (2017–2022), DRIVE demonstrated the possibilities and remaining challenges of brand-specific IVE evaluation across Europe, despite the hurdles encountered (mainly the need for larger sample sizes that were difficult to achieve due to competing networks and PPP concerns). DRIVE included more than 35,000 patients to collect data on ~60 variables and cover 13 different influenza vaccines, which conform to the DRIVE database. These data will be open for additional analysis and secondary use as part of the open-access framework for research.

The DRIVE project ended under the IMI umbrella in July 2022, and the EMA granted former DRIVE vaccine company partners a deferral on providing brand-specific IVE data for the 2022–2023 season. Consequently, the DRIVE consortium will not be conducting any influenza studies in the 2022–2023 season, although discussions with the EMA are ongoing to further define the future of DRIVE and IVE monitoring in Europe.

## Data availability statement

The datasets presented in this study can be found in online repositories. The names of the repository/repositories and accession number(s) can be found in the article/Supplementary material.

## Ethics statement

Each local study was approved by national, regional or institutional Ethics Committees, as appropriate. All the studies were approved without the need for revision of the protocols; therefore, the Ethics Committee clearance was given in due time for the start of the DRIVE study. Written informed consent was obtained in two-thirds of the participating study sites; in the remaining third of study sites (i.e., THL, MUV, GTPUH, EL, HVUH…), informed consent was not needed, as the DRIVE study was nested into the national/regional influenza surveillance systems.

## Collaborators

DRIVE Study contributors (group authorship):

- **NIID (Romania):** Anca Cristina Drăgănescu, Oana Săndulescu, Daniela Piţigoi, Victor Daniel Miron, Anca Streinu-Cercel, Anuţa Bilaşco, Adrian Streinu-Cercel, Dragoş Florea, Ovidiu Vlaicu, Simona Paraschiv, Leontina Bănică, and Dan Oţelea.
- **MUV (Austria):** Monika Redlberger-Fritz, Eva Geringer.
- **SUH (Spain):** Amparo López-Bernus, Ana Haro Perez, Nieves Gutierrez Zufiaurre, Cristina Carbonell Muñoz, Miguel Marcos Martin, Juan Luis Muñoz Bellido, Isabel Gil Rodríguez, Antonio Muro Alvarez, and Moncef Belhassen Garcia.
- **CIRI-IT (Italy):** Giancarlo Icardi, Stefano Mosca, Donatella Panatto, Emanuele Montomoli, Silvana Castaldi, Andrea Orsi, Alexander Domnich, Maria Chironna, Daniela Loconsole, Ilaria Manini, Christian Napoli, Alessandra Torsello, Elena Pariani, and Piero Luigi Lai.
- **HUVH and HUJT (Spain):** Susana Otero-Romero, Andrés Antón Pagarolas, Cristina Andrés, Ingrid Carbonés, Oleguer Pares, Mar Fornaguera, Anna Oller, Xavier Salgado, Patricia Tejerina, and Cristina Martinez.
- **FISABIO (Spain):** Alejandro Orrico-Sánchez, F. Xavier López-Labrador, Beatriz Mengual-Chuliá, Judit Sánchez Soler, María Jinglei Casanova Palomino, Juan Mollar-Maseres, Miguel Tortajada-Girbés, Noelia Rodríguez-Blanco, Mario Carballido-Fernández, Raquel Andreu Ivorra, Àngels Sierra Fortuny, Beatriz Segura Segura, Cristina Mingot Ureta, Sagrario Corrales Díaz-Flores, Ángela Sánchez Pla, María Dolores Tirado Balaguer, Juan Alberola, José Miguel Nogueira, Juan J Camarena, Francisco Arjona-Zaragozí, Maruan Shalabi Benavent, José Luis López-Hontangas, and María Dolores Gómez.
- **HULP (Spain):** Alejandro Martín-Quirós, Carlos Cañada Illana, and Emilio Cendejas.
- **Hospital Germans Trias i Pujol (Spain)**: Irma Casas García, Guillermo Mena Pinilla, María Esteve Pardo, Lola Álamo Junquera, Cristina Casañ, Sandra Fernandez Morodo, Agueda Hernández, Pere-Joan Cardona, Marta Segura, Andreu C. Pelegrin, Sara González-Gómez, Verónica Saludes, and Elisa Martró.
- **Directorate of Health (Iceland):** Valtýr Stefánsson Thors and Kristín L. Björnsdóttir.
- **I-REIVAC (France):** Liem Luong, Zineb Lesieur, Yacine Saidi, Rebecca Bauer, Christine Pereira, Philippe Vanhems, Fabrice Lainé, Florence Galtier, Xavier Duval, and Christine Durier.
- **University of Florence:** Paolo Bonanni, Alfredo Vannacci, and Claudia Ravaldi.

## Author contributions

AC and AS led and wrote the first draft of the manuscript. AD, UB, and ML reviewed the first draft and completed methods and results content accordingly. All authors are part of the DRIVE work package in charge of IVE studies and participated in the development of this manuscript, critically reviewed the drafts, provided feedback to complete the final version, which they read and approved, and had full access to the data. The material is original and has not been submitted elsewhere. The authors included in the group authorship collected the data reported in the present manuscript and critically reviewed the drafts and provided specific feedback.
