# Supplementary material for: Brand-specific estimates of influenza vaccine effectiveness for the 2021–2022 season in Europe: results from the DRIVE multi-stakeholder study platform
Source: Front Public Health. 2023 Jul 20;11:1195409. doi: 10.3389/fpubh.2023.1195409 (PMC10399959; doi:10.3389/fpubh.2023.1195409)
Supplement: Supplementary file 1 [file Data_Sheet_1.docx]

Supplementary Material

Brand-specific influenza vaccine effectiveness estimates during the 2021-22 season in Europe: results from the DRIVE multi-stakeholder study platform

Anke L. Stuurman^1*^, Antonio Carmona^2,3*^, Jorne Biccler^1^, Alexandre Descamps^4^, Miriam Levi^5^, Ulrike Baum^6^, Ainara Mira-Iglesias^2,3^, Stefania Bellino^7^, Uy Hoang^8^, Simon de Lusignan^8^, Roberto Bonaiuti^9^, Bruno Lina^10^, Caterina Rizzo^11^, Hanna Nohynek^6^, Javier Díez-Domingo^2,3^ and DRIVE Study Contributors^12^

**Correspondence:**Antonio Carmona
[acarmonaserrano1@gmail.com](mailto:acarmonaserrano1@gmail.com)

**Supplementary Methods**

***DRIVE generic protocols, statistical analysis plan and results reports***

DRIVE generic protocols are available at the DRIVE website [Deliverables section](https://www.drive-eu.org/index.php/results/deliverables/).

Data collected at the study contributors were anonymised and transferred to the DRIVE Research Server where they were analysed centrally by P95. IVE was only calculated if a pre-established minimum number of influenza cases (any influenza type/subtype, regardless of exposure) was available in the DRIVE dataset, stratified by setting (primary care, hospitals) and age group (6m-17yr, 18-64yr, ≥65yr). In case the threshold was met for a certain setting and age group, all the other analyses applying to this population (by influenza type and subtype, by influenza vaccine type and brand) would also be performed. For more details on the statistical methods, DRIVE Statistical Analysis Plan is available at the DRIVE website and is registered at the ENCePP EUPAS Register (EUPAS46888).

A study report was produced at the end of each season since 2017 and can be accessed through DRIVE website [Results section](https://www.drive-eu.org/index.php/results/).

Full results for the 2019/20 season onwards can be found in an interactive WebAnnex accessible at <https://apps.p-95.com/drivewebapp>.

***DRIVE sample size estimation***

Assuming a vaccine coverage of 50% (such as the observed among older adults in several countries with DRIVE sites in 2018/19), brand proportion of 25% and a VE of 40%, 625 cases would be required to yield crude IVE estimates with a precision of 40% and 300 of 60% in a brand-specific TND study with a 1:2 case-control ratio. To place this number in context, the total number of cases among older adults recruited in the hospital setting among older adults was 559 in 2018-19 and 304 in 2019-20. More details can be found at: <https://p-95.com/phdthesisanke/>


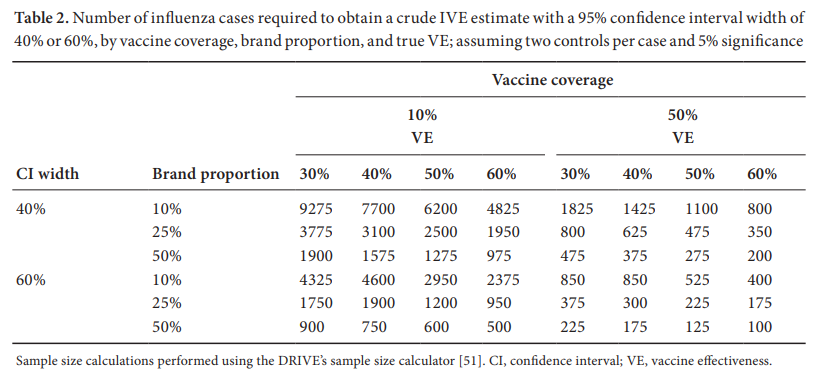


**Supplementary methods table 1.** Number of influenza cases required to obtain a crude IVE estimate with a 95% confidence interval width of 40% or 60%, by vaccine coverage, brand proportion, and true VE; assuming two controls per case and 5% significance. Sample size calculations performed using the DRIVE’s sample size calculator (<https://apps.p-95.com/app/drivesamplesize>). CI: confidence interval; VE: vaccine effectiveness

**Supplementary tables**

|  |  |  | **Influenza type/subtype** | | | | | |
| --- | --- | --- | --- | --- | --- | --- | --- | --- |
|  | **Number of tested samples for influenza*** | **All influenza cases (% of total tested samples)** | **A**  **n (% of total)** | **A(H1N1)pmd09**  **n (% of A with known subtype)** | **A(H3N2)**  **n (% of A with known subtype)** | **B**  **n (% of total)** | **B/Victoria**  **(%of B with known lineage)** | **B/Yamagata**  **(%of B with known lineage)** |
| **Austria** |  |  |  |  |  |  |  |  |
| **MUV** | 2427 | 171 (7) | 170 (99) | 6 (4) | 162 (96) | 1 (1) | NA | NA |
| **Finland** |  |  |  |  |  |  |  |  |
| **THL** | NA | 331 (NA) | 308 (93) | NA | NA | 23 (7) | NA | NA |
| **France** |  |  |  |  |  |  |  |  |
| **I-REIVAC** | 306 | 42 (14) | 42 (100) | 8 (53) | 7 (47) | 0 (0) | 0 (0) | 0 (0) |
| **Iceland** |  |  |  |  |  |  |  |  |
| **DH GP** | 296 | 96 (32) | 96 (100) | 0 (0) | 95 (100) | 0 (0) | 0 (0) | 0 (0) |
| **DH HOSP** | 1203 | 309 (26) | 309 (100) | 2 (1) | 307 (99) | 0 (0) | 0 (0) | 0 (0) |
| **Italy** |  |  |  |  |  |  |  |  |
| **CIRI-BIVE** | 317 | 15 (5) | 15 (100) | 0 (0) | 15 (100) | 0 (0) | 0 (0) | 0 (0) |
| **ISS** | 534 | 144 (27) | 144 (100) | 1 (1) | 142 (99) | 0 (0) | 0 (0) | 0 (0) |
| **Romania** |  |  |  |  |  |  |  |  |
| **NIID** | 141 | 77 (55) | 77 (100) | 3 (4) | 72 (96) | 0 (0) | 0 (0) | 0 (0) |
| **Spain** |  |  |  |  |  |  |  |  |
| **FISABIO** | 645 | 57 (9) | 57 (100) | 0 (0) | 55 (100) | 0 (0) | 0 (0) | 0 (0) |
| **GTPUH** | 71 | 34 (48) | 34 (100) | 0 (0) | 8 (100) | 0 (0) | 0 (0) | 0 (0) |
| **HUVH-HUJT** | 279 | 44 (16) | 44 (100) | 1 (3) | 36 (97) | 0 (0) | 0 (0) | 0 (0) |
| **LPUH** | 40 | 10 (25) | 10 (100) | 0 (0) | 0 (0) | 0 (0) | 0 (0) | 0 (0) |
| **SUH** | 58 | 40 (69) | 40 (100) | 0 (0) | 39 (100) | 0 (0) | 0 (0) | 0 (0) |
| **England** |  |  |  |  |  |  |  |  |
| **RCGP RSC** | 21 | 0 (0) | 0 (0) | 0 (0) | 0 (0) | 0 (0) | 0 (0) | 0 (0) |

NA: not available; *i.e. number of subjects retained for analysis

**Supplementary Table 1.** Description of the influenza cases included in the DRIVE dataset in the 2021-22 season, by type and subtype, for each study contributor

|  |  | **Influenza cases** | | | **Controls** | | | **Total** |
| --- | --- | --- | --- | --- | --- | --- | --- | --- |
| **Age group** | **SARS-CoV-2 test result** | **All, n (% of cases)** | **Vacc., n (% of vacc cases)** | **Unvac., n (% of unvacc cases)** | **All, n (% of cases** | **Vacc., n (% of vacc controls)** | **Unvac., n (% of unvacc controls)** | **n (% of total)** |
| **Primary care** | |  |  |  |  |  |  |  |
| 6m-17y | Pos. | 1 (0.5) | 0 (0.0) | 1 (0.6) | 352 (31.8) | 23 (13.2) | 329 (35.3) | 353 (27.3) |
|  | Neg. | 77 (40.7) | 7 (35.0) | 70 (41.4) | 510 (46.1) | 81 (46.6) | 429 (46.0) | 587 (45.3) |
|  | Mis. | 111 (58.7) | 13 (65.0) | 98 (58.0) | 244 (22.1) | 70 (40.2) | 174 (18.7) | 355 (27.4) |
| 18-64y | Pos. | 2 (1.1) | 0 (0.0) | 2 (1.3) | 635 (43.9) | 34 (18.2) | 601 (47.7) | 637 (39.1) |
|  | Neg. | 87 (48.1) | 6 (18.8) | 81 (54.4) | 566 (39.1) | 50 (26.7) | 516 (40.9) | 653 (40.1) |
|  | Mis. | 92 (50.8) | 26 (81.2) | 66 (44.3) | 247 (17.1) | 103 (55.1) | 144 (11.4) | 339 (20.8) |
| ≥65y | Pos. | 1 (2.4) | 0 (0.0) | 1 (4.2) | 132 (52.6) | 30 (35.7) | 102 (61.1) | 133 (45.5) |
|  | Neg. | 3 (7.3) | 2 (11.8) | 1 (4.2) | 61 (24.3) | 10 (11.9) | 51 (30.5) | 64 (21.9) |
|  | Mis. | 37 (90.2) | 15 (88.2) | 22 (91.7) | 58 (23.1) | 44 (52.4) | 14 (8.4) | 95 (32.5) |
| **Hospital** | |  |  |  |  |  |  |  |
| 6m-17y | Pos. | 3 (1.4) | 0 (0.0) | 3 (1.5) | 23 (3.9) | 0 (0.0) | 23 (4.2) | 26 (3.2) |
|  | Neg. | 43 (20.4) | 2 (25.0) | 41 (20.2) | 133 (22.6) | 12 (31.6) | 121 (22.0) | 176 (22.0) |
|  | Mis. | 165 (78.2) | 6 (75.0) | 159 (78.3) | 433 (73.5) | 26 (68.4) | 407 (73.9) | 598 (74.8) |
| 18-64y | Pos. | 15 (7.3) | 0 (0.0) | 15 (8.5) | 159 (23.5) | 26 (17.3) | 133 (25.2) | 174 (19.7) |
|  | Neg. | 86 (41.7) | 8 (27.6) | 78 (44.1) | 242 (35.7) | 42 (28.0) | 200 (38.0) | 328 (37.1) |
|  | Mis. | 105 (51.0) | 21 (72.4) | 84 (47.5) | 276 (40.8) | 82 (54.7) | 194 (36.8) | 381 (43.1) |
| ≥65y | Pos. | 7 (3.3) | 5 (3.6) | 2 (2.7) | 262 (22.1) | 118 (17.7) | 144 (27.8) | 269 (19.3) |
|  | Neg. | 81 (38.4) | 50 (36.2) | 31 (42.5) | 483 (40.8) | 284 (42.6) | 199 (38.4) | 564 (40.4) |
|  | Mis. | 123 (58.3) | 83 (60.1) | 40 (54.8) | 439 (37.1) | 264 (39.6) | 175 (33.8) | 562 (40.3) |

Mis.: missing; Neg.: negative; Pos.: positive; Vacc.: vaccinated with influenza vaccine; unvacc.: not vaccinated with influenza vaccine.

**Supplementary Table 2:** Number and percentage of subjects with a confirmed SARS-CoV-2 infection at the time of swabbing for influenza (only among those tested for SARS-CoV-2) identified in the DRIVE dataset for the 2021-22 season (TND studies), by influenza testing result and influenza vaccination status.

|  | IVE % [95% CI] against any influenza | | | | | | | |
| --- | --- | --- | --- | --- | --- | --- | --- | --- |
| Age | Efluelda | Fluad | Fluad Tetra | Fluarix Tetra | Flucelvax Tetra | Fluenz Tetra | Influvac Tetra | Vaxigrip Tetra |
| **Primary Care** |  |  |  |  |  |  |  |  |
| 6m-17 y* | n/a | n/a | n/a | -34 [-642, 76] | x | **64 [25, 83]** | -163 [-2964, 77] | 74 [-1, 94] |
| 18-64 y** | x | n/a | n/a | x | x | n/a | -13 [-302, 68] | 3 [-110, 56] |
| ≥ 65 y | x | 57 [-5032, 100] | x | x | x | n/a | x | **81 [22, 95]** |
| **Hospital** |  |  |  |  |  |  |  |  |
| 6m-17 y* | n/a | n/a | n/a | x | x | x | -208 [-2491, 63] | 58 [-16, 85] |
| 18-64 y** | x | n/a | n/a | x | 95 [-8, 100] | n/a | 23 [-1050, 95] | 50 [-92, 87] |
| ≥ 65 y | -28 [-1288, 88] | -62 [-312, 36] | -43 [-273, 46] | x | x | n/a | -268 [-911, -34] | 32 [-135, 81] |

* ≥ 2 y for Flucelvax Tetra and Fluenz Tetra; ≥ 3 y for Influvac Tetra. ** ≥ 60 y for Efluelda.

CI: confidence interval; n/a: not applicable because vaccine not licensed for age group; PC: primary care; x: no estimate available.

**Supplementary Table 3:** Pooled confounder-adjusted brand-specific vaccine effectiveness estimates against any influenza by age and by setting from TND studies, 2021-22.

|  |  |  | **Any influenza**  **IVE % [95%CI]** | **A**  **IVE % [95%CI]** |  |
| --- | --- | --- | --- | --- | --- |
| **Mixed setting** | |  | |  |  |
| **6m-6y** | |  | |  |  |
|  | Vaxigrip Tetra | 37 **[**-50, 73] | | 54 **[**-30, 84] |  |
|  | Fluenz Tetra (2-6y) | 25 **[**-21, 53] | | 40 **[**-3, 65] |  |
| **≥65y** |  |  | |  |  |
|  | Vaxigrip Tetra | 15 **[**-12, 36] | | 13 **[**-15, 34] |  |
| **Hospital setting** | |  | |  |  |
| **6m-6y** | |  |  |  |  |
|  | Vaxigrip Tetra  Fluenz Tetra (2-6y) | 51 **[**-304, 94]  -42 **[**-265, 45] | | - |  |
| **≥65y** | |  | |  |  |
|  | Vaxigrip Tetra | 16 **[**-27, 45] | | - |  |

**Supplementary Table 4:** Confounder-adjusted brand-specific influenza vaccine effectiveness against any influenza and influenza A, Finland THL population-based cohort, mixed setting and hospital setting, 2021-22.

**Supplementary figures**

a)


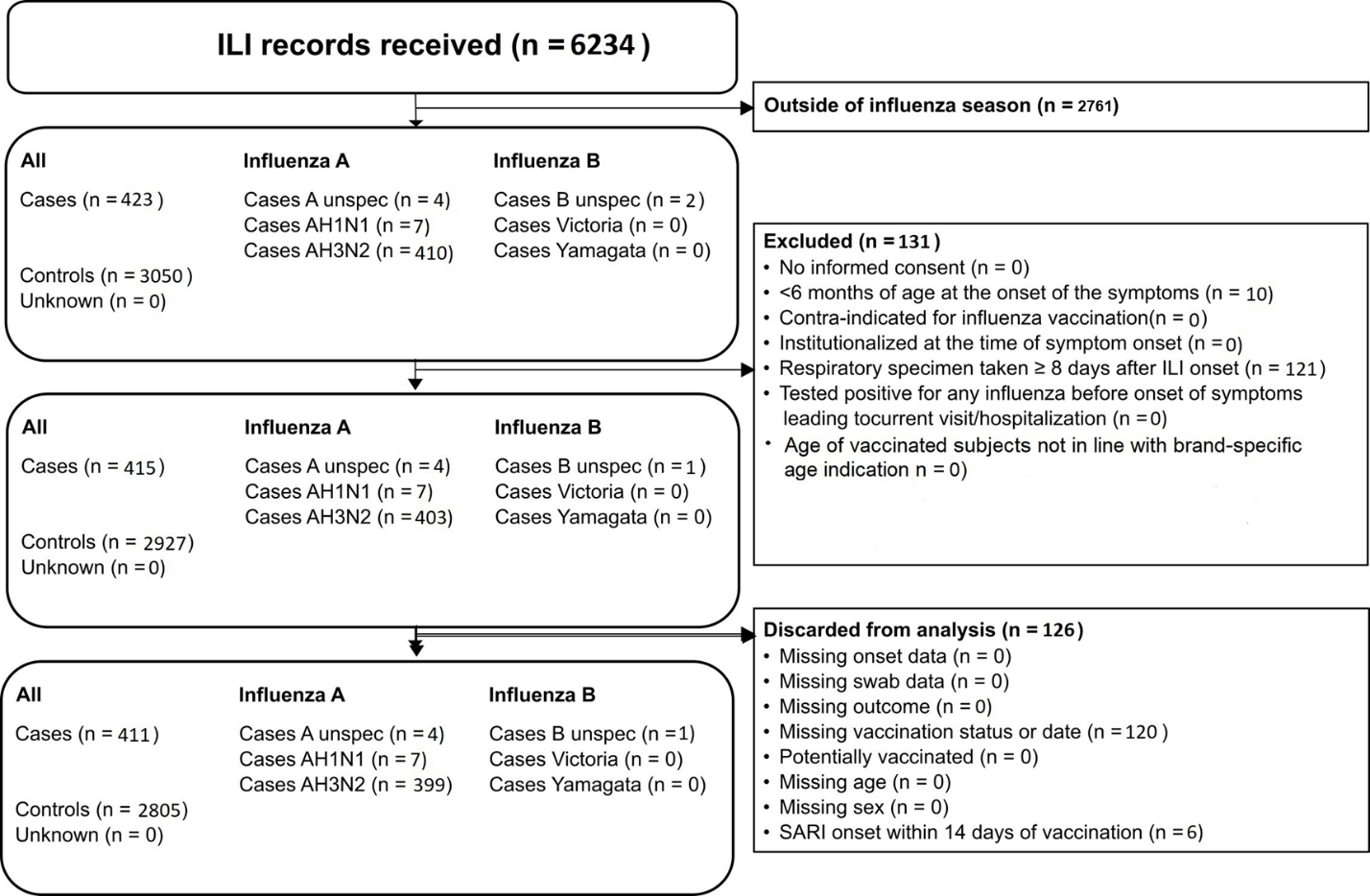


b)


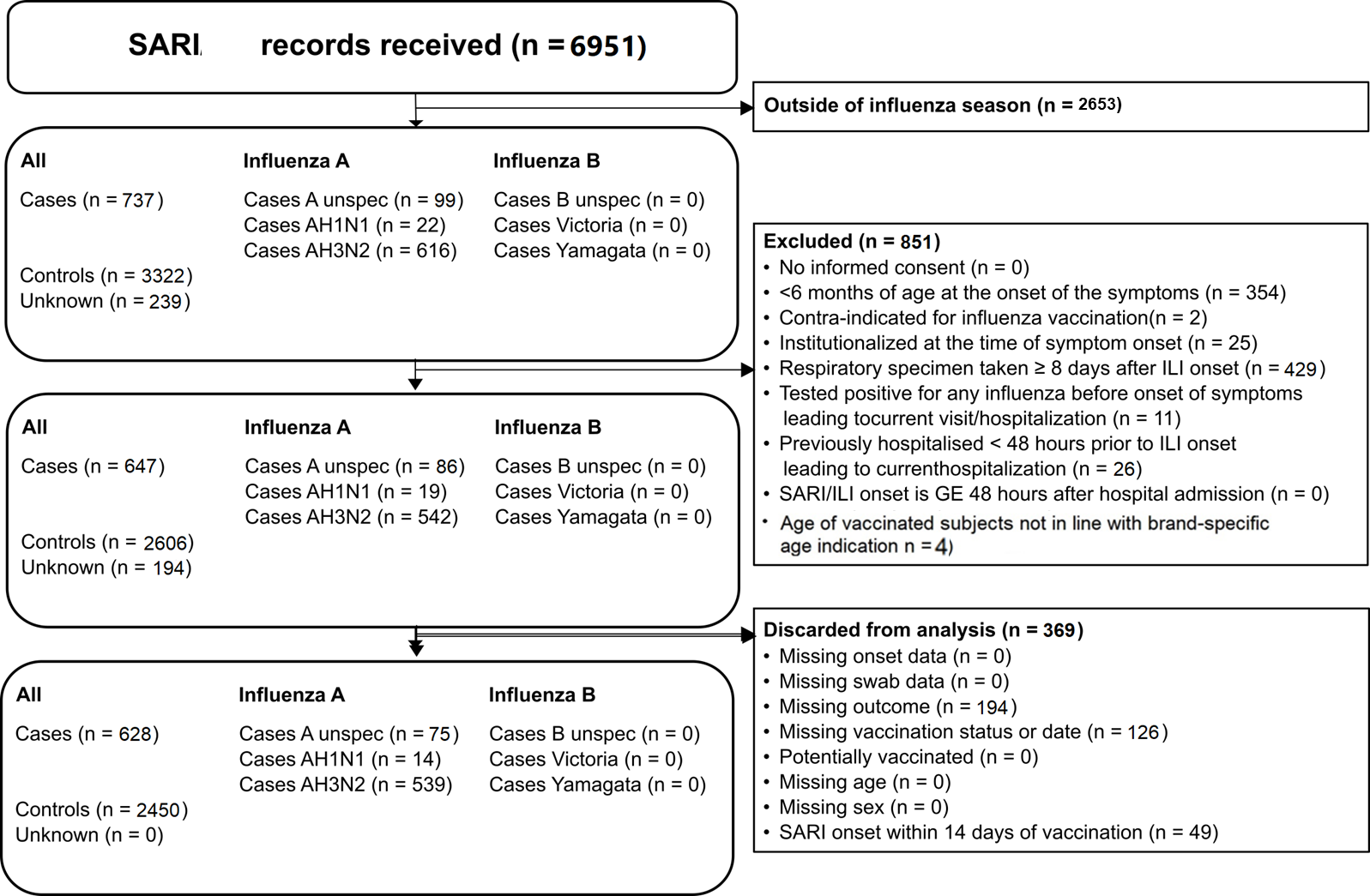


**Supplementary Figure 1:** Attrition diagrams by setting, TND studies pooled analysis. a) primary care setting (ILI subjects) and b) hospital setting (SARI subjects).


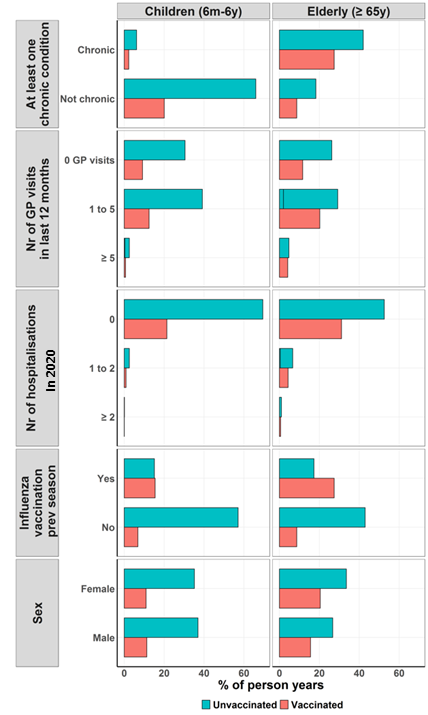


**Supplementary Figure 2:** Distribution of key covariates among exposed and unexposed recruited in the Finnish register-based cohort study, 2021/22.
